# Machine Learning Techniques for Differential Diagnosis of Vertigo and Dizziness: A Review

**DOI:** 10.3390/s21227565

**Published:** 2021-11-14

**Authors:** Varad Kabade, Ritika Hooda, Chahat Raj, Zainab Awan, Allison S. Young, Miriam S. Welgampola, Mukesh Prasad

**Affiliations:** 1 Department of Textile Technology, Indian Institute of Technology Delhi, New Delhi 110016, India; vkabade958@gmail.com; 2 Department of Computer Science and Engineering, Indian Institute of Technology Delhi, New Delhi 110016, India; ritika.hoodaa@gmail.com; 3 School of Computer Science, Faculty of Engineering and Information Technology, University of Technology Sydney, Sydney 2007, Australia; chahatraj58@gmail.com (C.R.); engr_zkawan@hotmail.com (Z.A.); 4 Central Clinical School, Faculty of Medicine and Health, University of Sydney, Sydney 2006, Australia; allison.young.audiologist@gmail.com (A.S.Y.); m.welgampola@gmail.com (M.S.W.); 5 Institute of Clinical Neurosciences, Royal Prince Alfred Hospital, Sydney 2006, Australia

**Keywords:** artificial intelligence, vertigo, dizziness, machine learning, feature extraction

## Abstract

Vertigo is a sensation of movement that results from disorders of the inner ear balance organs and their central connections, with aetiologies that are often benign and sometimes serious. An individual who develops vertigo can be effectively treated only after a correct diagnosis of the underlying vestibular disorder is reached. Recent advances in artificial intelligence promise novel strategies for the diagnosis and treatment of patients with this common symptom. Human analysts may experience difficulties manually extracting patterns from large clinical datasets. Machine learning techniques can be used to visualize, understand, and classify clinical data to create a computerized, faster, and more accurate evaluation of vertiginous disorders. Practitioners can also use them as a teaching tool to gain knowledge and valuable insights from medical data. This paper provides a review of the literatures from 1999 to 2021 using various feature extraction and machine learning techniques to diagnose vertigo disorders. This paper aims to provide a better understanding of the work done thus far and to provide future directions for research into the use of machine learning in vertigo diagnosis.

## 1. Introduction

**Figure 1:**
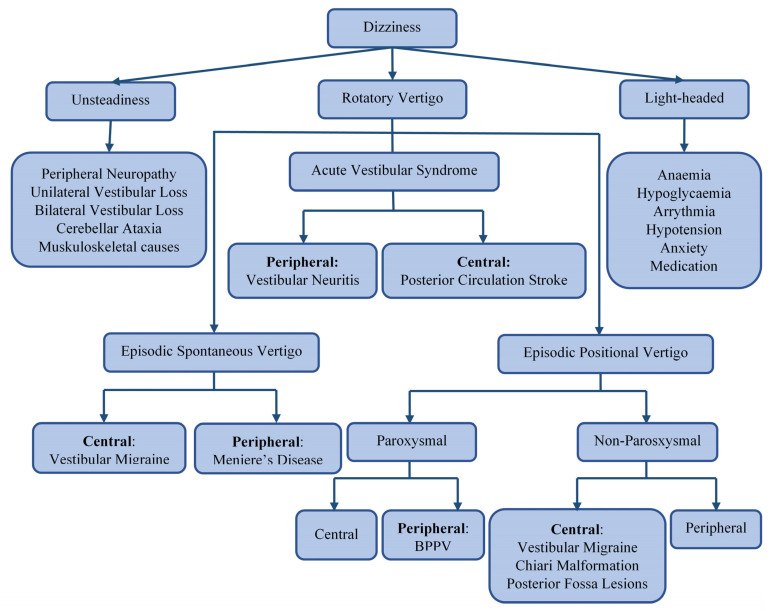
A flowchart depicting the differential diagnosis of dizziness.

Dizziness is a broad term that encompasses various symptoms, including unsteadiness, vertigo, and light-headedness or presyncope, as shown in Figure 1. Vertigo is an illusion of rotation, tilt, or any other movement of oneself (subjective vertigo) or one’s surroundings (objective vertigo) in any plane. It can be classified, by aetiology, into ‘peripheral’ or ‘central’, depending on the location of the dysfunction of the vestibular pathway. The vertigo that is caused by problems affecting the inner ear balance organs or vestibular end organs—the vestibular nerve or Scarpa’s ganglion—is classified as peripheral [1]. In contrast, vertigo that arises from disorders affecting the balance centers of the central nervous system (in the brain stem, cerebellum, or vestibular cortex) is classified as central [2]. Figure 1 illustrates common causes of different subtypes of dizziness. The most common causes of peripheral vertigo are benign paroxysmal positional vertigo (BPPV), Meniere disease (MD), and vestibular neuritis (VNE). The most encountered causes of central vertigo include vestibular migraine and posterior circulation stroke [3,4,5,6]. Since this classification is only applicable after a final diagnosis has been reached, a syndromic approach to vertigo has been adopted in clinical settings. Acute vestibular syndrome (AVS) refers to a single attack of severe vertigo lasting more than 24 h, with nausea and vomiting with postural instability, which is usually accompanied by spontaneous nystagmus [7]. Peripheral causes of AVS. include vestibular neuritis (VN), an innocuous viral inflammation of the vestibular nerve [8]. A posterior circulation stroke may cause central AVS. and is a life-threatening illness requiring urgent imaging and immediate treatment with oral antiplatelet and sometimes endovascular therapies [9]. Episodic spontaneous vertigo (ESV) refers to recurring attacks of spontaneous vertigo lasting more than 1 min, not triggered by changes in head movement [10]. One of the most common causes of ESV is vestibular migraine (VM), commonly presenting with spinning or swaying vertigo with a history of current or past migraines. Meniere disease (MD), a less common cause of ESV, is characterized by violent spinning vertigo, hearing loss, aural fullness, and tinnitus due to fluid build-up in the inner ear [11,12]. Episodic positional vertigo (EPV) refers to vertigo recurring due to changes in head position and is characterized by brief spells of spinning vertigo. EPV is most often due to benign paroxysmal positional vertigo (BPPV), caused by calcium carbonate particles dislodged into the semi-circular canals. Less commonly, EPV can be caused by vestibular migraine or other central disorders, such as posterior fossa tumors (central positional vertigo) [13,14].

Vertigo is a widespread and distressing symptom that may occur at any age. Dizziness (including vertigo) affects from about 15% to over 20% of adults yearly in extensive population-based studies [15]. Vestibular vertigo accounts for about a quarter of dizziness complaints and has a 12 month prevalence of 5% and an annual incidence of 1.4% [15]. In most affected individuals, vertigo results in a medical consultation, interruption of daily activities, or sick leave [16]. Prevalence and incidences increase with age and are reported much higher in women than in men [15]. In one study [17], the prevalence of vertigo and dizziness in people aged more than 60 years was found to be 30%. The presence of dizziness in the elderly can be a strong predictor of falls, leading to accidental death [18].

Vertigo, being a common symptom in the general population, merits an organized approach by healthcare practitioners at the levels of primary care, emergency room, and specialty services, in order to secure an early and accurate diagnosis. Misdiagnosis at the level of primary care or ER may result in inappropriate or ineffective therapies or referral to an incorrect specialist, thus prolonging the symptoms and increasing the likelihood of associated morbidities. Such a misdiagnosis is costly to the patient, the healthcare system, and the economy and is inefficient and potentially harmful. Neuhauser H.K. [15] suggests that BPPV and vestibular migraine (VM) are underdiagnosed, while MD is usually overdiagnosed. Dizziness and vertigo account for about 4% of presenting symptoms in the emergency department (ED) [19]. Stroke accounts for 4–15% of patients, and around 10% are missed at first contact, with a substantial increase in morbidity and mortality [20].

Recently, Ahmadi S.A. et al. [21] proposed that machine learning methods have the potential to perform better than clinical scores (e.g., HINTS (head impulse, nystagmus type, test of skew); ABCD2 (age, blood pressure, clinical features, duration of symptoms, diabetes)) in stroke detection. Machine learning (ML) has been widely used in disease diagnosis, with the aim of improving the speed and accuracy of diagnosis. ML has also been employed to analyze the importance of clinical parameters and their combinations for prognosis (e.g., prediction of disease progression), for the extraction of medical knowledge for outcomes research, therapy planning, and support, in order to improve overall patient management. Many attempts have been made to apply machine learning techniques for the differential diagnosis of vertigo over the last few decades. These models process the patients’ data, find the correlations and associations of presenting symptoms, familiar antecedents, habits, and background medical history with a view to predicting vertigo aetiology. The machine learning models most commonly used in vertigo diagnosis include decision trees [22,23,24,25], support vector machines (SVM) [22,25,26,27,28,29,30,31,32,33,34], k-Nearest neighbors (KNN) [20,23,25,26,27,30,35,36], and deep learning techniques [35,37,38]. Some researchers have also used novel ML algorithms and ensemble learning to improve diagnostic accuracy [28,33,39,40,41,42].

This paper aims to provide a comprehensive analysis of the application of artificial intelligence in the diagnosis of vertigo. The rest of the paper is organized as follows: Section 2 discusses different datasets and features used to train models. Section 3 discusses the advantages and disadvantages of ML techniques and explains the selection criteria of articles for the literature review. Section 4 provides a review of different ML techniques used in the literature, followed by a discussion in Section 5, with possible future directions for researchers, and finally, paper is concluded in Section 6.

## 2. Data Collection and Analysis for Machine Learning in a Specialist Vertigo Clinic

In a specialty clinic, the diagnostic process of the non-specific symptom of dizziness involves first eliciting information on the patient’s background medical history (comorbidities) and then clarifying presenting symptoms (whether the vertigo is spontaneous or positional, its duration, and associated phenomena). Next, a physical examination is performed—one important component of this examination is a general inspection for head tilt, obvious cranial nerve palsies, and Horner’s syndrome. Next, an eye examination, which includes assessment for spontaneous, gaze-evoked, and positional nystagmus, head impulse testing, testing saccadic and pursuit eye-movements, and assessment of vestibulo-ocular reflex suppression is undertaken. Tests of standing balance such as the matted Romberg test, Unterberger tests, tandem walking, and screening neurological tests for limb weakness and ataxia are important parts of the examination, as is postural blood pressure testing. In many instances, an expert clinician will arrive upon a diagnosis with the history and examination, in others, the assistance of audio vestibular tests—which interrogate the cochlear and vestibular end organ function—is sought. Tests conducted most often are audiometry to assess cochlear function, caloric testing, video head impulse tests to assess horizontal semi-circular canal function, and vestibular evoked myogenic potentials to test otolith function. These tests require interpretation in the context of the history and physical examination. Rotational chair testing is employed when seeking evidence of poor VOR suppression and for further quantification of bilateral vestibular loss (BVL). Posturography and gait analyses are now seldom used in the differential diagnosis of vertigo but can help identify vestibular disorders as a cause of imbalance, estimate the risk of falls, monitor the disorder’s progression, and track treatment effects [43]. Screening laboratory tests, such as a complete blood count, electrolytes, thyroid function tests, and vitamin B1 and B12 levels and iron studies, are used in seeking the nutritional and metabolic causes of dizziness. Imaging studies (MRI Brain, CT angiography, and CT Petrous temporal bones with canal plane reconstructions), seeking a structural cause for dizziness, are often undertaken. There are many tests that could be performed when assessing vertigo, yet their utility is context-specific, thus a clinician needs to select the tests as befits the clinical syndrome.

ONE [44] was an early expert system developed to aid the diagnosis of vertigo. It implemented a database of vertiginous patients for research purposes and used this database in several research studies [45,46]. The data included was collected through a questionnaire related to presenting symptoms, comorbidities, and results of vestibular, audiology, and imaging tests amounting to 170 variables. DizzyReg [47] is a modern clinical registry containing information on history, examination, test results, diagnosis, treatment, and outcome of patients with vertigo and dizziness. It contains anamnestic, sociodemographic, diagnostic, and therapeutic information of patients presenting with vertigo, including duration and type, neurological examination findings, audio vestibular test results, and the video head impulse test amounting to over 300 variables. The data was collected through the perusal of medical reports and questionnaires and used for intelligent diagnosis of vestibular disorders in [47]. The Dizziness Handicap Inventory (DHI) [33] is a validated questionnaire of twenty questions for quantitative evaluation of the degrees of handicap in the daily lives of patients with vestibular disorders. Data collected using DHI has been used for differential diagnosis of posterior canal benign paroxysmal positioning vertigo (PC-BPPV) and horizontal canal benign paroxysmal positioning vertigo (HC-BPPV) via machine learning techniques [33]. It is conceivable that a modern database created for diagnosis of vestibular disorders would consist of detailed history, physical examination, and laboratory test data, as well as expert diagnoses, treatments, and their outcomes, quantified by using validated questionnaires such as the Dizziness Handicap Inventory (DHI) or the Vertigo Symptom Scale (VSS) [25,48]. Once data are collected and pre-processed, a machine learning algorithm is chosen, and a model is trained on the data. A model to be used for diagnostic assistance should exhibit high sensitivity and high specificity.

## 3. Advantages and Disadvantages of Machine Learning Techniques and Selection Criteria of Articles for Literature Review

### 3.1. Advantages and Disadvantages of Machine Learning Techniques

This section discusses the advantages and disadvantages of various machine learning algorithms utilized in the existing literature for classifying vertigo types in Table 1. It includes support vector machines (SVM) [49,50], naïve Bayes (NB) [51,52], decision trees (DT) [53,54], K-nearest neighbors (KNN) [55,56], neural networks [57], and genetic algorithms [58].

### 3.2. Selection Criteria of Articles for Literature Review

**Figure 2:**
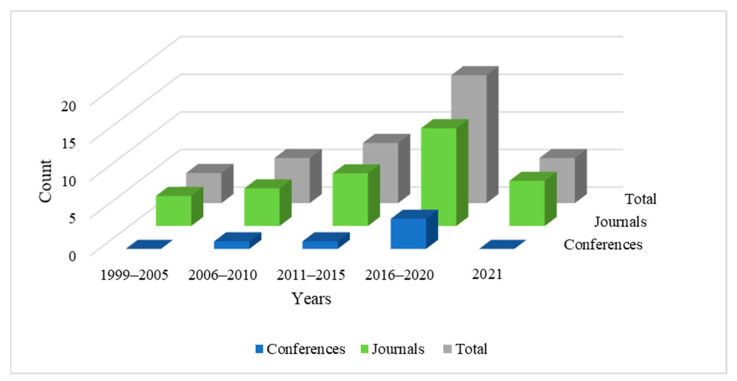
Number of articles published in journals and conferences from 1999 to 2021.

We conducted a targeted review of the scientific literature to examine and summarize articles that explore directions in artificial intelligence towards the diagnosis of vertigo and dizziness-related disorders. The search process involved defining a list of impactful journals and reputed databases, identifying search terms, and defining specific inclusion and exclusion criteria to filter out relevant articles for this study. The databases considered for this study are Scopus, Google Scholar, PubMed, IEEE Xplore, ScienceDirect, NCBI, ACM, Wiley, Taylor and Francis, and Springer Nature. Search terms included ‘machine learning and vertigo diagnosis’, ‘vertigo diagnosis and artificial intelligence’, ‘vertigo classification and machine learning’, ‘vertigo diagnosis and data mining’, ‘vertigo data analysis’, and ‘vertigo and dizziness diagnosis’. Synonyms and various combinations of these terms were also used to conduct an exhaustive search. The primary criteria of shortlisting relevant articles were to include all the studies involving the diagnosis of vertigo-related disorders using artificial intelligence. We have included all the papers employing machine learning, shallow learning, and deep learning for diagnosing vertigo-related disorders. We have excluded research articles that employ artificial intelligence for medical purposes other than the diagnosis of the disease. Articles focusing on artificial intelligence for clinical management and treatment of vertigo-related disorders have been excluded from this review; additionally, editorials, book reviews, and literature surveys were not considered. We studied over 100 research papers from the above-mentioned digital libraries and shortlisted 41 articles that we deemed relevant for this review. Thirty-five articles among these are published in highly reputed journals and six are from international conferences. We have reviewed articles published between the years 1999 and 2021. Figure 2 illustrates the year-wise trend of relevant articles published in this domain. It is notable that the number of published works has increased since 1999 and the research domain is gaining considerable significance, which motivated us to study this area. 

## 4. Machine Learning Approaches Used in the Differential Diagnosis of Vertigo

This section reviews the existing literature on machine learning approaches used for vertigo classification. We follow a feature-based approach instead of the traditional technology-based approach to organize the literature. This is due to the sparsity of literature in the usage of machine learning algorithms. Fellow researchers have employed multiple varying algorithms on specific target problems. It is easier to map the applications and group them together according to the types of data or features used, or both. Organizing the literature in accordance with algorithm usage makes it relatively difficult due to the huge number of existing algorithms, which leaves us with a greater number of subsections with fewer articles focusing on a given algorithm. Feature-based organization broadly arranges the literature into four major types of features studied or employed for vertigo classification, making the arrangement comprehensible and compact. The feature-based approach that we followed allowed us to understand the ongoing development in individual sub-domains and threw light on the algorithms already being employed for specific features, paving the way for future directions. Section 4.1 examines the literary sources that implemented machine learning algorithms on dataset ONE. Section 4.2 elaborates on the works utilizing questionnaire-based features, while Section 4.3 discusses the techniques using nystagmus features, and finally, Section 4.4 reviews the literature that uses posturography and gait features for vertigo identification.

### 4.1. Machine Learning Applications on ONE Dataset

ONE [44], an early expert system for vertigo, was developed as a diagnostic aid to assist teaching and implement a research database. The database of ONE consisted of patients’ responses to 170 questions relating to symptoms, background medical history, and vestibular test findings. A method based on pattern recognition was used in the reasoning process with attribute weights initially set by neuro-otology experts. Kentala E. et al. [59] developed Galactica, a genetic algorithm approach to discover differential diagnostic rules to classify data into six dissimilar vestibular disorders (BPV, Meniere disease, sudden hearing loss, vestibular neuritis, vestibular schwannoma, traumatic vertigo) taken from ONE’s database. Their proposed genetic algorithm developed IF–THEN rules from the questionnaire dataset, annotated as positive or negative. The authors used a sample size of 200 patients, setting 150 as the number of generations and keeping 0.95 and 0.01 as the probabilities of crossover and mutation, respectively. The accuracy of the rules was above 90% for all diseases except Meniere disease, for which the accuracy level was 81%. Varpa K. et al. [60] noted that combining machine-learnt weights with expert knowledge gave the best classification results, classifying 82.5–84.7% of cases correctly within the first and second diagnostic suggestions.

Missing data limits the applicability of various machine learning algorithms. Data imputation is used to overcome this drawback, where missing values in continuous data are replaced by the mean or median of the specific feature and replaced by the mode in categorical values. The results achieved by machine learning after imputation depend on the size of missing values, where accuracy decreases with the rise in the number of missing items. Laurikkala et al. [61] studied the usefulness of different imputation techniques such as means, regression, expectation maximization, and random imputation to treat missing values in this dataset, to allow for multivariate statistical analysis. They found that the discriminant functions obtained from imputed data were highly accurate for all the methods (93–96%). Their findings indicated that the missing data did not adversely affect disease classification. Miettinen et al. [62] employed Bayesian methods on the ONE database to classify diseases accurately. Bayesian probabilistic models could also reveal dependence relations between attributes used for classification. Juhola et al. [23] compared KNN, discriminant analysis, k-means clustering, decision trees, multi-layer perceptron (MLP) networks, and Kohonen networks on this data, after data preprocessing with principal component analysis (PCA). Linear discriminant analysis performed the best, followed by MLP networks.

Varpa K. et al. [63] compared attribute weighting methods with decision support system ONE and one-vs.-all (OVA) KNN classifiers to classify nine vertiginous diseases (see Table 2). The best total accuracy was achieved with the attribute-weighted 5-nearest neighbor OVA method using the scatter weights. Varpa K. et al. [64] used a genetic algorithm-based approach for attribute weighting in ONE, class weighted KNN, and OVA weighted KNN, which improved disease classification accuracy, median, and true positive rates of all the methods. Varpa K. et al. [30] compared one-vs.-all and one-vs.-one methods in KNN and SVM and found that using multiple binary classifiers (one-vs.-one) improved the true positive rates of disease classes. Joutsijoki H. et al. [65] used half-against-half (HAH) architecture with SVM, KNN, and naïve Bayes (NB) methods with HAH–SVM reaching similar accuracy as OVO–SVM. Juhola M. et al. [66] tested the classification capability of neural networks on ONE database. Since the data had unbalanced distribution, MLP and Kohonen networks could detect the big classes with high specificity but failed to detect the smaller classes. Siermala M. et al. [31] creates a set of neural networks for each disease class and artificial cases for smaller classes. It was found that this methodology could successfully deal with class imbalance, giving high classification accuracy even for smaller classes. Shilaskar et al. [32] dealt with a class imbalance on this dataset by synthetic oversampling of minority class and under-sampling of majority class and using modified PSO algorithm for feature selection and SVM for improving accuracy.

Table 2 summarizes the results for studies done on the dataset ONE, showing the mean accuracy (Acc.), mean specificity (Spec.), mean sensitivity (Sens.), and mean F1 score (F1) for all target classes. The other notations used in the Table 2 are as follows: VS—vestibular schwannoma; BPPV—benign paroxysmal positional vertigo; MD—Meniere disease; SD—sudden deafness; TV—traumatic vertigo; VNE—vestibular neuritis; VES—vestibulopatia; BRV—benign recurrent vertigo; CL—central lesion; ANE—acoustic neuroma.

### 4.2. Machine Learning Applications to Questionnaire-Based Information and Multi-Feature of DHI and DizzyReg Dataset

Machine learning and statistical techniques applied to other questionnaires and multi-feature databases containing relevant information about the patients’ history, vestibular test findings, symptoms, etc., have also been used for creating intelligent diagnostic systems [21,24,33,37,39,40,43,45,67,68]. Ahmadi, S.A. et al. [21] compared machine learning approaches on multi-feature data sets (including a standardized assessment of symptom features, cardiovascular risk factors, and detailed quantitative testing of ocular motor, vestibular, and postural function) vs. clinical scores such as HINTS, ABCD2 for differential diagnosis of vestibular stroke, and vestibular neuritis. Logistic regression, random forest, artificial neural network (ANN), and geometric matrix completion (e.g., Single or MultiGMC) were used where MultiGMC outperformed clinical scores. Random forest was used to rank features based on their discriminative power to understand the diagnosis better. 

Masankaran, L. et al. [33] use the DHI questionnaire to distinguish between BPPV types. Recursive feature elimination and feature importance with extra trees classifier selected the Gaussian naïve Bayes classifier that gave the best performance, with 73.91% accuracy. Grözinger, M. et al. [37] used deep neural networks trained on DizzyReg [47] for diagnosing vestibular migraine and Meniere disease in clinical practice. Strobl, R. et al. [67] used classification and regression trees to diagnose eight different vestibular disorders based on only eight critical variables from the DizzyReg dataset. Kim, B.J. et al. [68] compared various classification models such as SVM, logistic regression, and random forest Catboost to differentiate between central and non-central causes of dizziness by using simple clinical information such as demographics, medical history, systolic and diastolic blood pressure, and heart rate. Additionally, the Shapley additive explanations (SHAP) value was used to explain the importance of each variable in the clinical information for diagnosis. Exarchos, T.P. et al.’s study [24] consists of a recommendation tool to guide the general practitioners and experts and a diagnostic model for 12 balance disorders. It uses wrapper feature selection methods with decision trees enhanced with AdaBoost trained on a dataset with 350 features containing detailed patient information to create 1 binary model each for 12 different balance disorders. Richburg, H. et al. [39] suggests a survey-based support system for diagnosing BPPV, which does not require a physician interfacing with the software. It uses attribute selection filters and wrappers and decision trees on patient data collected through a questionnaire. Rasku, J. et al. [45] created a computerized peer support system for Meniere disease program that can verify and assess the diagnosis of Meniere disease by using a pattern recognition method.

The dynamic uncertain causality graph (DUCG) [36] is a newly proposed probabilistic graphical model, which can deal with systems with logic cycles, dynamics, and uncertainties. Dong, C. et al. [40] proposes a novel diagnostic and reasoning modeling to identify between 22 etiologies, based on investigations and relevant characteristics of vertigo using DUCG methodology. Dong, C.-L. et al. [43] uses a DUCG based differential diagnostic model for subtype differentiation of benign paroxysmal positional vertigo (BPPV). The symptoms, signs, findings of examinations, medical histories, etiologies, and pathogeneses are incorporated in both diagnostic models. They manifest higher diagnostic correctness than other ML-based methods, good robustness to incomplete medical data, and provide a rationale for choosing a disease.

Table 3 shows the results for studies done on other questionnaires and multimodal databases showing mean accuracy (Acc.), mean specificity (Spec.), mean sensitivity (Sens.), and mean F1 score (F1) for all target classes. The other notations used in Table 3 are as follows: CV—cross validation; BPPV—benign paroxysmal positional vertigo; MD—Meniere disease; VNE—vestibular neuritis; CL—central lesion; ANE—acoustic neuroma; VP—vestibular paroxysmia; VM—vestibular migraine; t-BPPV—typical benign paroxysmal positional vertigo; a-BPPV—atypical benign paroxysmal positional vertigo; PPPD—persistent postural perceptual dizziness; UPD—unilateral peripheral dysfunction; BVD—bilateral vestibular dysfunction; AVS—acute vestibular syndrome nystagmus; UVP—unilateral vestibulopathy; BVP—bilateral vestibulopathy, FD—functional dizziness.

### 4.3. Machine Learning Applications to Nystagmus and Vestibulo-Ocular Reflex (VOR) Tests

Nystagmus is defined as an involuntary rapid and rhythmic movement of the eyeball and is often associated with vertigo. Amine, B.S. et al. [29] proposed a videonystagmography (VNG)-based machine learning approach to identify vestibular neuritis. These investigators video recorded nystagmus, used a pupil tracking algorithm to measure nystagmus metrics, and then used Fischer criteria for feature selection and SVM for classification, which gave classification results higher than K-nearest neighbor and artificial neural networks with an accuracy of 94.1%. BPPV can affect any one of the three semicircular canals, but most often affects the posterior or horizontal canal which can be identified with nystagmus patterns. Lim, E.C. et al. [35] used a deep learning model trained on extracted image data from nystagmus videos induced by positional tests to classify the affected canal in BPPV patients. More recently, a novel deep learning based framework involving convolutional neural networks was introduced for automatic detection of torsional up beating nystagmus of PC BPV from nystagmus videos [38]. When tested on a clinically collected torsional nystagmus video dataset, the method showed promising results in frame-level identification of torsional motion and final torsional nystagmus segment localization, which can help clinicians improve their diagnostic accuracy. Juhola M., et al. [25] used a signal analysis technique on video head impulse tests which assess the vestibulo-ocular reflex to differentiate healthy subjects from those with vestibular loss affecting the semicircular canals. These investigators sought to separate controls from acoustic neuroma and used KNN, linear discriminant analysis, naïve Bayes, SVM, k-means clustering, MLP network, Kohonen network, and decision trees, with decision trees yielding the best accuracy of 89.8%.

Table 4 shows the results for studies done on nystagmus data showing mean accuracy (Acc.), mean specificity (Spec.), mean sensitivity (Sens.), and mean F1 score (F1) for all target classes. The other notations used in the Table 4 are as follows: CV—cross validation; VNE—vestibular neuritis; ANE—acoustic neuroma; PC-BPPV—posterior canal benign paroxysmal positional vertigo; HC-BPPV—horizontal canal benign paroxysmal positional vertigo; T-BPPV—torsional canal benign paroxysmal positional vertigo.

### 4.4. Machine Learning Applications to Posturography and Gait Features

To diagnose disorders related to human balance systems, clinicians sometimes use methods of recording body sway. Machine learning techniques applied to posturography and gait analysis parameters could potentially aid the diagnosis of balance disorders. Pradhan, C. et al. [26] used pattern recognition techniques on posturography and spatiotemporal gait data of 150 samples acquired on a gait mat to identify gait disorders. SVM and ANN differentiated the gait patterns with higher sensitivity and specificity compared with KNN and NB. SVM reported highest results with 93% sensitivity and 97% specificity. Ahmadi, S.A. et al. [27] used static posturography signal patterns for automatic classification into eight disorders, including Parkinson’s disease, phobic postural vertigo, acute vestibular syndrome, and cerebellar disorders. KNN, SVM, ANN, logistic regression, random forest, and extra forest were used for classification where extra forest performed better than others. An ensemble method (stacking classifier) combining all these classifiers gave the best performance. The t-distributed stochastic neighbor embedding (t-SNE) technique was used to plot the data into two dimensions that showed clear clusters of diseases. Ikizoglu, S. et al. [28] compared two feature selection techniques (T-test and sequential backward selection) and two feature transformation techniques (principal component analysis and kernel principal component analysis with Gaussian and polynomial kernels) for dimensionality reduction on data obtained from dynamic posturography to be used with SVM. Feature transformation techniques resulted in more accurate models, and dimensionality reduction helped in reducing the computation time.

Heydarov, S. et al. [22] compared SVM, SVM with Gaussian kernel, and decision tree on gait data to classify vestibular system disorders and found SVM with Gaussian kernel to perform better than others. Krafczyk et al. [69] used ANN to classify four neurological and vestibular disorders, based on static posturography characteristics with high overall sensitivity. Zhang et al. [48] proposed an SVM-based method for determining gait disturbances of BPPV by collecting data in clinical settings with the help of wearable accelerometers. The data was collected from 27 outpatients and 27 healthy subjects by observing different temporal–spatial and gait-specific variables while they walked wearing the sensor. The data collected was used for training SVM with a linear kernel with 5-fold cross-validation. This study suggested that wearable technologies are an excellent source for collecting data that can be used to train ML models for diagnosing vertigo and related illnesses. Such technologies, interfaced with smart devices, are easier to integrate within users’ everyday routines and collect information at regular intervals. They reduce the need of clinical tests, offering the users a remote environment to record their health data. Kamogashira, T. et al. [70] used ensemble algorithms such as gradient boosting classifier, bagging tree on center of pressure (COP) sway during foam posturography measured from patients with dizziness, to predict vestibular dysfunction. Gradient boosting achieved 82% sensitivity followed by random forest and logistic regression with 81% and 78% sensitivity, respectively.

Table 5 shows the results for studies done on posturography and gait data showing mean accuracy (Acc.), mean specificity (Spec.), mean sensitivity (Sens.), and mean F1 score (F1) for all target classes. The other notations used in the Table 5 are as follows: VNE—vestibular neuritis; ANE—acoustic neuroma; PPV—phobic postural vertigo; CA—cerebellar ataxia; BV—bilateral vestibulopathy; PSP—progressive supranuclear palsy; OT—orthostatic tumor; DN—downbeat nystagmus; AVS—acute unilateral vestibulopathy; PD—Parkinson’s disease; PNP—poly-neuropathy.

## 5. Discussion and Potential Directions

Could the integration of artificial intelligence into medical diagnosis significantly improve the speed and accuracy of diagnosing vestibular disorders? The answers are unclear for several reasons, as follows: (1) Investigators have sometimes sought to answer a given diagnostic question without using the highest yield data (for example, diagnosing BPV with a questionnaire or with posturography, when nystagmus profiles provide the answer). (2) Seeking to separate large numbers of disorders rather than a few differential diagnoses for a single presentation. (3) Modern laboratory tests that diagnose specific vestibular disorders (vHIT for vestibular neuritis [71], ictal nystagmus for Meniere disease [72], VEMP for superior canal dehiscence [73]) have not been used in ML endeavors. The merits of increased AI usage in medical diagnosis include the following: (1) bringing machine learning expertise where human expertise is unavailable; (2) reducing manual tasks and the freeing up of a given physician’s time; (3) increasing efficiency and productivity by providing a scalable application.

Kim, B.J. et al. [68] applied ML algorithms on simple clinical information such as demographics and medical histories, obtained at early stages or emergency centers, can perform a differential diagnosis for vertigo disorders. Such algorithms, once optimized, could assist non-expert physicians treating vertigo in the frontline. There is a scope of using embedded systems with trained ML models to help in the early diagnosis of acute vertigo. Attribute weighting and selection methods, Bayesian networks, dynamic, uncertain casualty graphs, decision trees, and random forests can help assess the relative importance of attributes for disease classification [40,43,62]. Lim, E.C. et al. [35] suggest the possibility of using a deep learning architecture embedded on any device that can record eye movement to classify nystagmus types into subtypes of BPPV directly. Filippopulos et al. [74] suggests using an AI-based, computerized clinical decision support system with an easy-to-use mobile application and systematic expert support to improve diagnostic accuracy and outcomes of patients presenting with acute vertigo syndromes in primary care.

Existing studies that have utilized machine learning algorithms have highlighted the limited availability of large-size clinical datasets [39,46]. Small sizes of clinical datasets and missing values in clinical records, such as demographics, medical history, and results from clinical tests tend to reduce the performance of machine learning algorithms. Training on large clinical datasets is imperative for machine learning models to yield robustness and high classification results. High-dimensional data with multiple types of features such as demographics, patients’ medical histories, several clinical test results, increases the search space, and algorithmic computation time. Various irrelevant features that do not contribute as an identification factor of a disease among such high-dimensional data need to be identified and excluded to reduce the feature-set dimension. Few studies have focused on feature extraction and feature transformation methods to reduce the feature-set dimension, achieving increased classification accuracy, also preventing overfitting [23,28,29]. The machine learning techniques in existing literature provide an automated procedure for disease prediction by interpreting complex clinical data, mainly resorting solely to model selection and parameter determination. It is important to focus on the underlying pathogenesis and pathophysiology instead of solely relying on machine learning classification models. Future studies should consider merging the intelligent diagnostic system with the physician’s interpretation in clinical medicine.

**Figure 3:**
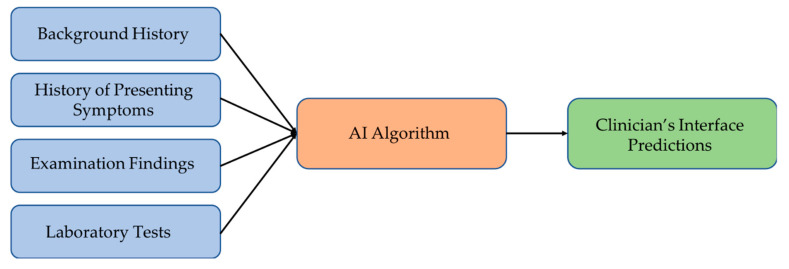
Suggested vertigo disorders diagnostic framework.

The studies suggest that there is a need to develop a decision support system (DSS) that can cover a wide range of vertiginous diseases [39,45,68], which should be able to collect the input data into a database that may be later used to retrain models and improve accuracy. Figure 3 illustrates the suggested model of such a decision support system for disease diagnosis. The AI algorithm of the system maps the input data to the most plausible diagnosis. An ensemble of different machine learning models should work better than individual classifiers for predicting the disease. The DSS should also handle cases with incomplete clinical evidence either through extrapolating from the previous database or using methods capable of working with incomplete inputs, thereby achieving robustness and higher accuracies in vertigo classification.

## 6. Conclusions

Vertigo is a common symptom arising from many etiologies, ranging from benign to potentially severe. This paper summarizes the use of modern artificial intelligence techniques in the differential diagnosis of acute vertigo. Despite the long history of using AI for neuro-otological diagnoses, a superior diagnostic support system has not yet emerged. Publicly available datasets of patients with diverse vertigo presentations and the results of their interrogation with new, widely available audiovestibular tests are likely to encourage future researchers to undertake much-needed work in this domain.

## Figures and Tables

**Table 1:** Advantages and disadvantages of algorithms commonly used for vertigo classification.

Algorithm	Advantages	Disadvantages
Decision Trees	Requires less pre-processing, does not need normalization and scaling, no need of data imputation	Instability, complex calculations, high training time, resource expensive, and does not work with continuous values
Support Vector Machines	Efficient with distinctive classes, high dimensional data spaces, memory efficient, excellent for few data samples	Poor performance with large datasets, sensitive to noise and overlapping classes, underperforms when no. of features >no. of samples
K-Nearest Neighbor	No training period, faster execution, supports dynamic data addition, needs only two parameters	Poor performance with large datasets, inefficient with high dimensional data, scaling, and normalization required, sensitive to noise, outliers, and missing values
Naïve Bayes	Time inexpensive, supports multi-label classification, needs less training samples, best suited for categorical data	Less applicability in real-life scenarios due to feature independence, assigns zero probability to missing values
Genetic Algorithms	Highly accurate, provides optimal results, robust and straightforward	Computation expensive, requires high parameter optimization
Neural Networks	Automatic feature extraction, robust to data variations, scalable to large data volumes, adaptive to varying problems	Requires large training set, resource expensive, high computation time, complex to comprehend and optimize

**Table 2:** Performance of machine learning algorithms on ONE dataset.

Year	ML Algorithm	Target	Sample Size	Evaluation	Performance			
					Acc.	Sens.	Spec.	F1
2000 [61]	Discriminant analysis and regression imputation	VS vs. BPPV vs. MD vs. SD vs. TV vs. VNE	564	176 test cases	95	95	90	-
2008 [23]	k-nearest neighbor (k = 5)	VS vs. BPPV vs. MD vs. SD vs TV vs. VNE	815	10-fold CV	93.5	85.45	-	-
	Linear discriminant analysis				95.5	91.81	-	-
	K-means clustering (k = 20)				92.9	84.83	-	-
	Decision trees				89.4	71.45	-	-
	Multi-layer perceptron network				95.0	90.46	-	-
	Kohonen network				92.7	82.95	-	-
2008 [31]	Perceptron neural networks	ANE vs. BPPV vs. MD vs. SD vs. TV vs. VNE vs. BRV vs. CL vs. VES	815	815	95	85	83	-
2010 [62]	Naïve Bayes	ANE vs. BPPV vs. MD vs. SD vs. TV vs. VNE	815	10-fold CV	97	90	-	-
	tree augmented naïve Bayes				97	89	-	-
	General Bayesian network				97	91	-	-
2011 [30]	k-nearest neighbor (k = 5)	ANE vs. BPPV vs. MD vs. SD vs. TV vs. VNE vs. BRV vs. CL vs. VES	1030	10-fold CV	79.8	77.9	-	-
	One-vs.-one support vector Machine-linear				77.4	82.4	-	-
	One-vs.-one k-nearest neighbor (k = 5)				82.4	88.2	-	-
	One-vs.-all support vector machines-rbf				79.4	78.6	-	-
	One-vs.-all k-nearest neighbor (k = 5)				78.8	77.7	-	-
2013 [65]	Half and half Support vector Machine-linear	ANE vs. BPPV vs. MD vs. SD vs. TV vs. VNE vs. BRV	1030	10-fold CV	76.9	-	-	-
	Half and half k- nearest neighbor (k = 9)				61.5	-	-	-
	Half and half naïve Bayes				65.9	-	-	-
	Multinomial logistic regression				68.3	-	-	-
2014 [64]	Genetic algorithm class weighted k-nearest neighbor (k = 9)	ANE vs. BPPV vs. MD vs. SD vs. TV vs. VNE vs. BRV	951	10-fold CV	68.8	64.1	-	-
	Genetic algorithm One-vs.-all weighted k-nearest neighbor (k = 3)				79.5	74.9	-	-
2016 [32]	Feed forward neural networks	ANE vs. BPPV vs. MD vs. SD vs. TV vs. VNE	815	5-fold CV	84	84	97	84
	Grid-based SVM				91	91	98	91
	Forward feature selection-based SVM				90	90	98	90
	Genetic algorithm-based SVM				92	92	98	92
	Modified PSO algorithm-based SVM				94	94	99	94
2017 [63]	Weighted one-vs-all k-nearest neighbor (k = 5)	ANE vs. BPPV vs. MD vs. SD vs. TV vs. VNE vs. BRV	1030	10-fold CV	79.7	75.2	-	-

**Table 3:** Performance of machine learning techniques on questionnaires and multi-features of DHI and DizzyReg dataset.

Year	ML Algorithm	Target	Sample Size	Evaluation	Performance			
					Acc.	Sens.	Spec.	F1
2016 [24]	Decision trees enhanced with Adaboost and expert knowledge	ANE vs. t-BPPV vs. a-BPPV vs. VP vs. VM vs. MD vs. CL vs. PPD vs. VNE vs. UPD vs. BVD vs. others	985	10-fold CV	82.65	81.61	83.6	-
2018 [39]	Decision tree	BPPV	45	45 training cases	92	88	95	-
2018 [33]	Gaussian naïve Bayes	BPPV	114	10-fold CV	73.91	-	-	72.73
	K-nearest neighbor (k = 11)				69.57	-	-	69.68
	Support vector machines—poly				65.22	-	-	64.53
	Random forest				65.22	-	-	65.35
2020 [37]	Deep neural networks	VM vs. MD	346	10-fold CV	98.2	87.65	-	-
	Boosted decision trees				88.9	63.9	-	-
2020 [21]	Logistic regression	Vestibular stroke vs. Peripheral AVS	40	5-fold CV	52	-	-	-
	Multi-geometric matrix completion				82	-	-	-
2021 [68]	Support vector machine	Central vs. non-central dizziness	3116	1310 test cases	-	99.2	11.6	-
	Logistic regression				-	99.2	6.8	-
	Random forest				-	99.2	6.0	-
	Catboost				-	100	4.6	-
2021 [67]	Classification and regression trees	MD vs. BPPV vs. VM vs. UVP vs. BVP vs. VP	1066	10-fold CV	42.2	-	-	-

**Table 4:** Performance of machine learning techniques on nystagmus and vestibulo-ocular reflex tests.

Year	ML Algorithm	Target	Sample Size	Evaluation	Performance			
					Acc.	Sens.	Spec.	F1
2008 [25]	K-nearest neighbor (k = 5)	ANE	44	10-fold CV	87.7	79.2	94.2	-
	Linear discriminant analysis				87.6	81.1	92.5	-
	Quadratic discriminant analysis				87	84.9	88.6	-
	Naïve Bayes				88.3	82.7	92.5	-
	K-means clustering (k = 2)				85	78.2	90.2	-
	Decision trees				89.8	83.6	94.7	-
	Multi-layer perceptron networks (16 hidden nodes)				88.8	82.9	93.4	-
	Kohonen networks 7 × 7 nodes				87.6	78.9	94.2	-
	support vector machines (radial)				89.4	82.7	94.6	-
2019 [29]	K-nearest neighbor	VNE	60	5-fold CV	85.3	86.5	87.6	-
	Artificial neural network				86.8	88.3	89.5	-
	Fischer-support vector machine				94.1	93.2	95.9	-
2019 [35]	Convolutional neural network	PC-BPPV vs. HC-BPPV vs. T-BPPV	3457	10-fold CV	-	80.8	97.1	79.4
2021 [38]	Convolutional neural network	T-BPPV	8000	8:2 Train–Test Split	85.7	78.9	-	81.0

**Table 5:** Performance of machine learning techniques on posturography and gait features.

Year	ML Algorithm	Target	Sample Size	Evaluation	Performance			
					Acc.	Sens.	Spec.	F1
2006 [69]	Artificial neural network	PV vs. CA vs. VNE	60	60 validation cases	-	93	93	-
2015 [26]	Artificial neural network	PPV vs. CA vs. PSP vs. BV	150	10-fold CV	-	90.6	96.1	-
	Support vector machine				-	93	97	-
	K-nearest neighbor				-	73.3	92.3	-
	Naïve Bayes				-	77	93.8	-
2017 [22]	Support vector machine	Vestibular system disorders vs. healthy	18	5-fold CV	75	-	-	-
	Support vector machine with Gaussian kernel				81.3	-	-	-
	Decision tree				62.5	-	-	-
2019 [27]	K-nearest neighbor	OT vs. PPV vs. CA vs. DN vs. AVS. vs. PNP	293	50-fold CV	64.5	-	-	-
	Extra forest				80.7	-	-	-
	Stacked classifier				82.7	-	-	-
2020 [28]	Support vector machine–polynomial	Vestibular system disorders vs. healthy	37	-	81.0	-	-	-
	Support vector machine–Gaussian				89.2	-	-	-
2020 [70]	gradient boosting classifier	Vestibular dysfunction vs. healthy	238	5- fold CV	-	82	-	-
	Logistic regression				-	78	-	-
	Random forest				-	81	-	-

## Data Availability

Not applicable.
